# Similarity‐Enhanced Representation Learning of Non‐Canonical Amino Acids for Therapeutic Peptide Modeling

**DOI:** 10.1002/advs.77511

**Published:** 2026-09-01

**Authors:** Chencheng Xu, Lesong Wei, Jianmin Wang, Yuanpeng Xiong, Ruochi Zhang, Yu Wang, Chao Zha, Qiangcheng Zeng, Xin Gao

**Affiliations:** ^1^ Syneron Opal George Town Cayman Island; ^2^ School of Mechanical Engineering Tongji University Shanghai China; ^3^ Department of Integrative Biotechnology Yonsei University Incheon Republic of Korea; ^4^ Department of Life Science Dezhou University Dezhou China; ^5^ Computer Science Program, Computer, Electrical and Mathematical Sciences and Engineering Division King Abdullah University of Science and Technology (KAUST) Thuwal Saudi Arabia; ^6^ Center of Excellence for Smart Health (KCSH) King Abdullah University of Science and Technology (KAUST) Thuwal Saudi Arabia; ^7^ Center of Excellence on Generative AI King Abdullah University of Science and Technology (KAUST) Thuwal Saudi Arabia

**Keywords:** amino acid, computational biology, computer science, encode, feature learning, graph, in silico, peptide, small molecule

## Abstract

Peptides combine the favorable pharmacokinetics of small molecules with the high specificity of biologics, making them promising therapeutics. Incorporating non‐canonical amino acids (ncAAs) further enhances drug‐like properties, yet modeling remains challenging due to chemically modified residues and combinatorial sequence diversity. Here, we introduce SinCAA, a similarity‐enhanced pretraining framework specifically designed to encode ncAAs. The framework is built on the principle that amino acids with similar 3D conformations induce minimal perturbations to peptide properties. It jointly optimizes two complementary self‐supervised tasks: contrastive learning guided by a conformational similarity metric to capture functional relationships among ncAAs, and masked node reconstruction to encode the unique chemical identity of each ncAA. Built on a graph transformer backbone, this dual “relationship–identity” supervision enables SinCAA to learn robust atomic representations that generalize from individual ncAA building blocks to full‐length peptides. SinCAA exhibits strong zero‐shot performance in peptide property prediction and consistently outperforms state‐of‐the‐art pretrained models across diverse benchmarks. This framework provides an efficient and interpretable approach for in silico prediction and ranking of ncAA‐containing peptides, accelerating candidate screening in therapeutic peptide discovery.

## Graphical abstract

Non‐canonical amino acids (ncAAs) enhance peptide therapeutics but remain difficult to model computationally. SinCAA, a similarity‐enhanced pretraining framework, jointly optimizes contrastive learning guided by a novel conformational similarity metric with masked node reconstruction, capturing both functional relationships and chemical identity of ncAAs. This approach enables efficient, interpretable prediction and ranking of ncAA‐containing peptides for drug discovery.

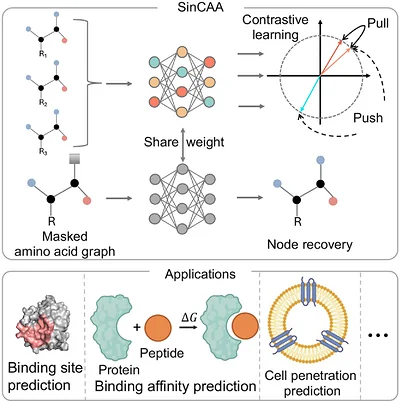

## 1 Introduction

Since the synthesis of insulin in 1921, more than 80 peptide drugs have been approved worldwide, making the development of novel therapeutic peptides one of the most active areas in pharmaceutical research [1]. Peptides typically consist of a linear sequence of amino acids, usually fewer than 50, representing a class of therapeutics that bridge the gap between small molecules and large biologics such as antibodies. They combine the favorable pharmacokinetic properties and membrane permeability often associated with small molecules with the high specificity and biological activity characteristic of larger protein‐based therapeutics. Examples of well‐known peptide drugs include abaloparatide for osteoporosis in postmenopausal women at high fracture risk [2], glucagon‐like peptide 2 (GLP‐2) [3] analogues for short bowel syndrome, and human glucagon‐like peptide 1 (GLP‐1) [4] for type 2 diabetes mellitus.

A major stumbling block in therapeutic peptide development is that peptides composed solely of natural amino acids often exhibit poor membrane permeability and in vivo stability [5, 6]. Non‐canonical amino acids (ncAAs) have thus been introduced to improve peptide properties. ncAAs are amino acid variants beyond the standard 20 used in ribosomal protein synthesis, enabling enhanced functional diversity and the design of cyclic peptides [7, 8, 9, 10]. The development of therapeutic peptides containing ncAAs remains challenging for several reasons. First, the synthesis of ncAAs and their incorporation into peptides remain challenging. The preparation of ncAAs is often labor‐intensive and low‐yielding, while their incorporation through genetic code expansion or solid‐phase peptide synthesis can suffer from limited efficiency, fidelity, and yield [11, 12, 13, 14, 15]. A second challenge lies in the vast sequence space introduced by incorporating ncAAs. Even adding just a few dozen ncAAs leads to a combinatorial explosion in sequence diversity. This renders experimental screening prohibitively time‐consuming and costly, especially considering that there could theoretically be tens of thousands of different ncAAs [16, 17].

Computational approaches for peptides containing ncAAs are primarily based on molecular dynamics simulations [8, 9]. Although these methods can provide detailed structural insights, they are computationally intensive and often require substantial expertise to ensure reliable results. A few machine learning methods have been proposed to predict the cell‐penetrating ability of cyclic peptides containing ncAAs, but they typically rely on extensive hand‐crafted features [18, 19], which may limit their scalability and generalizability. Machine learning frameworks for protein–peptide binding prediction are generally trained on structures from the Protein Data Bank (PDB) [20], many of which contain ncAAs. However, most of these models represent peptides as linear sequences defined over the canonical amino acid alphabet, with ncAAs represented as generic placeholders. This formulation fails to encode the chemical structures of ncAAs and is not directly applicable to cyclic peptides [21, 22, 23]. A fundamental limitation in developing machine learning models for peptides with ncAAs is the scarcity of high‐quality training data. Publicly available examples of such peptides are very limited, and for many ncAAs, there may be only a single occurrence across all known datasets. Consequently, existing peptide pretraining models [24] exhibit poor performance and cannot be effectively applied to general peptide property prediction tasks.

To address these challenges, we introduce SinCAA, a similarity‐enhanced pretraining framework for modeling therapeutic peptides containing ncAAs. Existing molecular similarity metrics, primarily designed for small molecules, fail to capture the subtle structural relationships among ncAAs that influence peptide conformation and function. We therefore develop a physicochemically informed similarity metric based on 3D conformational relationships, assuming that substituting an amino acid with a conformationally similar residue minimally perturbs the peptide's conformation and function. This metric captures the impact of even minor side‐chain modifications on amino acid conformation. Leveraging this metric, SinCAA jointly optimizes two complementary self‐supervised objectives: (i) contrastive learning aligns ncAAs with similar conformations in latent space, capturing functional relationships among chemically diverse residues; (ii) masked node reconstruction requires the model to infer missing atoms from local context, learning the atomic composition and bonding patterns unique to each molecule. Implemented using a graph transformer, this dual “relationship–identity” supervision produces robust representations that generalize from ncAA building blocks to full‐length therapeutic peptides. We demonstrate the practical impact of SinCAA in drug discovery through multiple downstream tasks, including peptide–protein binding affinity prediction, cell‐penetrating ability estimation, and general protein–peptide interaction modeling. Compared to models pretrained on small molecules, SinCAA achieves a relative improvement of more than 10% in Spearman's ρ across all tested setting and exhibits strong zero‐shot capabilities, enabling rapid in silico screening of ncAA‐containing peptide libraries. These results establish SinCAA as an effective framework for prioritizing peptide candidates with favorable pharmacokinetic properties, helping to reduce the experimental burden of candidate screening in therapeutic peptide development.

## 2 Results

### 2.1 Similarity‐Enhanced Pretraining on Non‐Canonical Amino Acids

**FIGURE 1:**
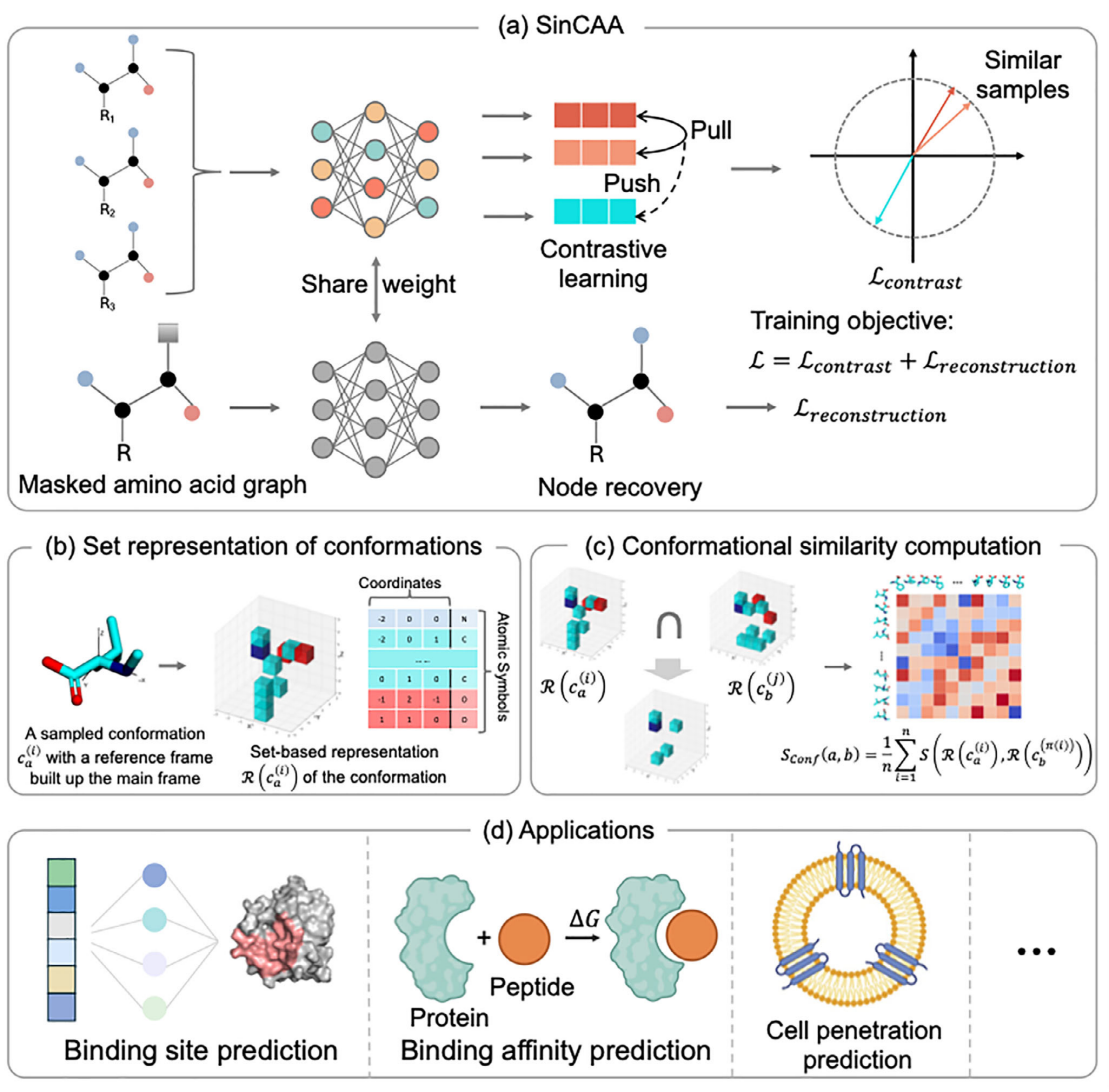
Overview of the SinCAA framework and the similarity metric for ncAAs. (a) Schematic of the SinCAA framework. ncAAs were curated from PubChem and used to pretrain a graph‐based transformer model. Pretraining involves two tasks: (1) identifying structurally similar ncAAs, where similarity is defined by conformational distributions, and (2) masked node recovery on molecular graphs, augmented with molecules from ZINC15 to enhance the model's chemical representation learning. (b) Set‐based representation of molecular conformations. For a molecule *a*, the coordinates of each 3D conformation $c_{a}^{\left(i\right)}$ are standardized against a reference frame, then discretized into a voxel grid. Occupied voxels are recorded to form a set $R(c_{a}^{\left(i\right)})$. (c) Definition of conformational similarity between ncAAs. For each ncAA, *n* conformations are sampled and converted to voxel sets. Pairwise Jaccard similarity $S(R\left(c_{a}^{\left(i\right)}\right),\;R\left(c_{b}^{\left(j\right)}\right))$ is computed between conformations $c_{a}^{\left(i\right)}$ and $c_{b}^{\left(j\right)}$. The Earth Mover's Distance (EMD), calculated via the Hungarian algorithm to find the optimal alignment π*(*i*), defines the overall similarity *S_conf_* (*a*,*b*). (d) Generalizability of SinCAA. The framework can be applied to multiple tasks, including peptide binding site prediction, peptide binding affinity prediction, and peptide cell penetration prediction.

The overall framework of SinCAA is depicted in Figure 1. A total of 387 482 ncAAs were curated from PubChem [25], preprocessed, and randomly partitioned into training and validation sets. The pretraining stage involves two tasks: node recovery and contrastive learning, as shown in Figure 1a. The contrastive learning task enables the model to capture similarity relationships between amino acids, guided by a novel similarity measure specifically designed for amino acid structures. In parallel, the node recovery task adopts the masked‐node prediction strategy commonly used in molecular pretraining [26, 27, 28], where a subset of atoms in the molecular graph is randomly masked and the model is trained to recover their identities. As the number of ncAAs remains relatively limited for training an efficient model, the node recovery task was supplemented with molecules from the ZINC15 database [29] to enhance the model's ability to extract chemical information.

The amino acid similarity metric, termed conformational similarity, is based on the 3D conformations and chemical compositions of amino acids and is illustrated in Figure 1b–c. Starting from SMILES representations, 3D conformations are generated using the ETKDG [30] algorithm in RDKit, which has been shown to provide reliable conformer generation for drug‐like molecules of comparable size [31]. Only 0.86% of these ncAAs contain elements outside of the common set (C, H, N, O, S, P, F, Cl, Br, I), indicating that the vast majority of ncAAs in our dataset share the elemental composition commonly encountered in ETKDG‐parameterized molecules. To approximate the chemical changes associated with peptide bond formation, each amino acid was capped with glycine residues at both the N‐ and C‐termini during ETKDG conformer generation. This provides a simplified representation of the intrinsic, context‐independent conformational preferences of an ncAA after incorporation into a peptide. It does not capture the specific conformation that the residue would adopt within a particular macrocyclic peptide. To enable consistent spatial comparisons, a reference coordinate system is established for each conformation by centering the α‐carbon (Cα) at the origin, aligning the nitrogen atom from the amino group along the negative x‐axis, and defining the xy‐plane using the nitrogen and carboxyl carbon atoms. This setup is similar to that used in AlphaFold [32]. Each 3D conformation is then discretized into a set‐based representation by gridding space and recording occupied voxels along with their atomic symbols and properties (Figure 1b). The similarity between two conformations is defined as the Jaccard similarity between their respective sets. Each voxel is labeled with elemental type and partial charge to preserve chemical information beyond spatial occupancy. To compare two ncAAs, which may each adopt multiple conformations, we treat their conformations as distributions and approximate the Earth Mover's Distance using the Hungarian algorithm to optimally match sampled conformations (Figure 1c). In downstream applications (Figure 1d), given the molecular graph of a peptide, SinCAA generates embeddings for each atom, which can be used for various predictive tasks, including peptide binding site prediction, protein–peptide binding affinity prediction, and peptide cell‐penetration prediction.

The core assumption underlying the use of conformational similarity is that substituting amino acids with similar 3D conformations tends to introduce smaller perturbations to peptide structures, a principle widely used in peptide engineering [9, 33]. Traditionally, peptide conformational similarity is characterized using backbone dihedral angle distributions (ϕ/ψ), such as Ramachandran‐based analyses. Our conformational similarity provides an efficient and scalable surrogate for angle‐based similarity. Given the limited availability of experimental data on ncAA substitution effects, we performed a proof‐of‐concept analysis of nine amino acid substitutions in a cyclic peptide from a single published dataset [9] (Figure S1 and Table S1). Amino acid substitutions involving more conformationally similar residues resulted in smaller perturbations in binding affinity (Kd), yielding a Spearman correlation of 0.83 for affinity ranking, compared with 0.60 for Morgan fingerprint similarity. The results in Figure S2 further show that conformational similarity better distinguishes the stereochemical differences among ncAAs, whereas Morgan fingerprint similarity offers substantially weaker discrimination. To incorporate this principle into representation learning, we employed contrastive learning, focusing on relative rather than absolute similarity. For each amino acid, we randomly sampled two candidates from its top 20 most similar ncAAs and assigned labels based on their relative conformational similarity: the more similar candidate served as the positive pair and the less similar one as a hard negative, encouraging the model to embed conformationally similar amino acids closer while learning fine‐grained distinctions among near neighbors. We utilize an in‐batch negative sampling strategy, where all other samples within the same batch are treated as negatives. Given the size of our ncAA training set (309 986 molecules), the probability of false negatives is low, and the sparse noise introduced by such cases is expected to have limited impact on representation learning. This is also consistent with the robustness of InfoNCE‐based contrastive learning to moderate false‐negative rates.

A key feature of SinCAA is its ability to generalize from small ncAA molecular graphs to large peptide molecular graphs. Several strategies were employed to achieve this. First, we used a data augmentation technique by concatenating multiple ncAAs into chains. Since ncAAs are the building blocks of peptides, this approach not only enlarges the pretraining dataset but also increases the likelihood of peptide *d*‐patterns occurring during training, improving generalization of graph neural networks [34] to larger molecular graphs. Analysis in Figure S3 shows that *1*‐ and *2*‐patterns of ncAAs and peptides in downstream tasks have high overlap, concatenating multiple ncAAs substantially improves overlap for higher‐order patterns (*3*‐ and *4*‐patterns). To balance performance with training cost, we selected one‐ and two‐ncAA concatenations for pretraining.

Second, the SinCAA architecture is based on the Graph Position‐aware Structure (GPS) Transformer [35], with a Graph Isomorphism Network (GIN) [36] as the local message‐passing encoder. To expand the model's receptive field for local structures, we employed two GIN layers per transformer block instead of the commonly used single layer [37]. Experimental results in subsequent sections demonstrate that this modification significantly enhances downstream performance. As shown in Figure S4, pretrained SinCAA accurately distinguishes conformationally similar ncAA pairs from randomly selected non‐similar pairs in the validation set, achieving AUROC values above 0.996, indicating that the model effectively learns the conformational similarity relationships encoded by our metric.

### 2.2 Prediction of Binding Properties for Macrocyclic Peptides Targeting KRAS

To evaluate whether a model pretrained exclusively on ncAAs, without exposure to full peptides, can generalize to larger peptide structures in therapeutic peptide discovery, we tested SinCAA on predicting the binding properties of macrocyclic peptides targeting KRAS. RAS proteins are small GTPases whose activity is regulated by binding GDP (inactive state) or GTP (active state). Persistent RAS activation plays a critical role in cancer progression, with KRAS being the most frequently mutated isoform in human cancers, particularly pancreatic, colorectal, and lung tumors [9, 38]. KRAS has long been considered “undruggable” due to the lack of suitable small‐molecule binding pockets [38].

The dataset was curated from the publicly available patent US20240158446A1 [39] and contains 2761 peptides with experimentally measured binding metrics, reflecting realistic scenarios in peptide drug design. Each peptide contains 10–12 amino acids, including 795 monocyclic peptides and 1966 peptides containing more than one cycle; cycles are defined as ring structures with at least eight atoms. We predicted two binding‐related properties: the dissociation constant (Kd), which quantifies peptide‐target binding affinity, and the half‐maximal inhibitory concentration (IC50), which reflects the concentration required to inhibit KRAS activity by 50% and can be influenced by factors such as cellular uptake. All peptides in this dataset bind to the same KRAS pocket [9, 33], so predictions were based on peptide topology alone, without explicitly modeling KRAS. The property distributions of the dataset are shown in Figure S5.

**FIGURE 2:**
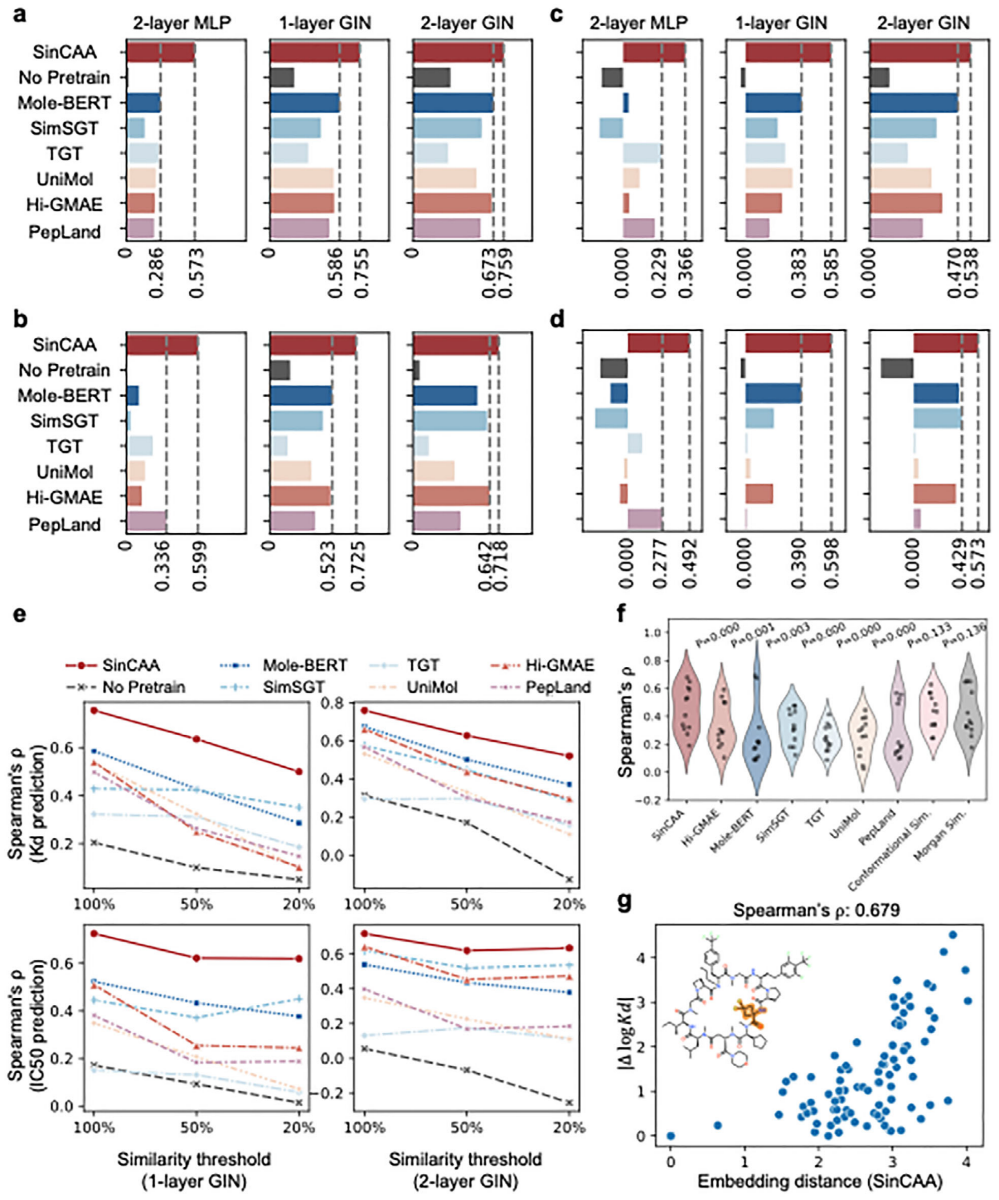
Prediction of binding affinities for macrocyclic peptides using SinCAA. (a, b) Comparison of SinCAA with baseline methods in predicting binding affinities measured by Kd (a) and IC50 (b). Across both metrics, SinCAA consistently outperforms existing molecular pretraining approaches when paired with a simple MLP or shallow GIN, achieving relative improvements in Spearman's ρ of more than 10% over the strongest baseline in all settings. (c, d) Performance on test sets containing ncAAs absent from training data. SinCAA surpasses all baselines, demonstrating robustness to unseen chemical space. (e) Prediction accuracy on test samples stratified by Morgan fingerprint Tanimoto similarity to the training set, where similarity is defined as the average similarity of a test peptide to all training peptides. SinCAA maintains strong performance for peptides with low similarity to the training set. (f) Spearman's correlations between representation‐based distances (or molecular similarities) and binding affinity differences for peptide groups containing single‐position substitutions. Each point represents one peptide group. Paired t‐test p‐values comparing each baseline method with SinCAA are shown. (g) Example peptide group containing single‐position substitutions. The substituted residue position is highlighted. Correlations between embedding distances and Kd changes are shown for different representation methods.

Since general protein‐pretrained models are not readily applicable to cyclic peptides, and existing approaches for protein–peptide binding prediction cannot adequately encode peptide cyclic topology or the chemical structures of ncAAs, we benchmarked SinCAA against state‐of‐the‐art small‐molecule pretraining approaches, as well as PepLand, a peptide‐specific pretrained model. As shown in Figure 2a–b, using either a simple MLP or a one‐/two‐layer GIN, SinCAA substantially outperformed existing models for both Kd and IC50 prediction, achieving over 10% improvement in Spearman's correlation compared to the best baseline. While most pretrained models outperform the no‐pretraining baseline, indicating that models trained on small molecules can partially capture relevant properties of ncAA‐containing peptides, their performance remained limited. This highlights fundamental differences between small molecules and cyclic peptides that SinCAA is specifically designed to address.

We next evaluated SinCAA's generalization. As shown in Figure 2c–d, test‐set peptides containing at least one amino acid absent from the training data were used to compare all methods. SinCAA consistently outperformed all baselines in Spearman's ρ. Notably, with a two‐layer MLP for IC50 prediction, most baseline models exhibited negative correlations with experimental values, indicating poor generalization. In contrast, SinCAA achieved a Spearman's ρ of 0.492 under the same setting. When stratifying the test set according to the average Tanimoto similarity between each test peptide and the training set, computed using Morgan fingerprints (Figure 2e and Figure S6), SinCAA maintained strong performance even among the 20% least chemically similar peptides. Together, these results highlight its robust generalization to peptides containing novel amino acids and exhibiting low chemical similarity to the training set.

A key challenge in therapeutic peptide design is the scarcity of labeled data due to high experimental costs, motivating the development of zero‐shot modeling approaches. To evaluate this capability, we constructed peptide groups differing by single‐residue substitutions, mimicking single‐residue scanning experiments commonly used in wet‐lab peptide engineering. We evaluated whether distances in the embedding space correlate with experimentally measured changes in Kd following these substitutions. As shown in Figure 2f,g and Figure S7, SinCAA embeddings exhibited stronger zero‐shot correlations with Kd changes than all baseline embeddings, with statistically significant improvements based on paired t‐tests. SinCAA embeddings also slightly outperformed Morgan fingerprint similarity in this task, achieving an average Spearman's ρ of 0.452 compared with 0.421 for Morgan similarity, although the difference was not statistically significant (paired t‐test, p = 0.136). Comparison between conformational similarity and Morgan similarity (Figure S8) showed that conformational similarity achieved higher performance in 7 of 12 groups and significantly improved prediction of Kd differences relative to the lead peptide, defined as the variant with the highest binding affinity within each substitution group. Together, these results demonstrate the utility of SinCAA embeddings and the proposed conformational similarity metric for zero‐shot estimation of peptide binding affinity changes.

Finally, ablation studies assessed the contributions of the similarity metric and ncAA pretraining data. We varied the number of appended amino acids during data augmentation, the number of GIN layers in each GPS module, the inclusion of contrastive loss, and the use of ncAAs in pretraining. As shown in Table S2, pretraining with ZINC15 alone achieved moderate performance. Incorporating ncAAs, using two GIN layers per GPS module, and including the contrastive loss substantially improved performance, whereas the effect of varying the number of appended ncAAs was comparatively modest and differed across downstream architectures and prediction tasks. Prediction errors stratified by the number of ncAAs unseen during pretraining are shown in Figure S9. The errors did not increase monotonically with the number of unseen ncAAs, suggesting that model performance is not solely dependent on memorization of individual ncAAs observed during pretraining. This is expected as pretraining is performed at the amino acid level, whereas downstream tasks involve full peptide structures.

### 2.3 Prediction of General Therapeutic Peptide Properties

In addition to binding affinity, other critical properties of therapeutic peptides include target selectivity and cell‐penetrating ability. Target selectivity directly impacts the safety profile of peptide‐based drugs by minimizing off‐target effects, while cell‐penetrating ability determines whether a peptide can effectively reach intracellular targets.

**FIGURE 3:**
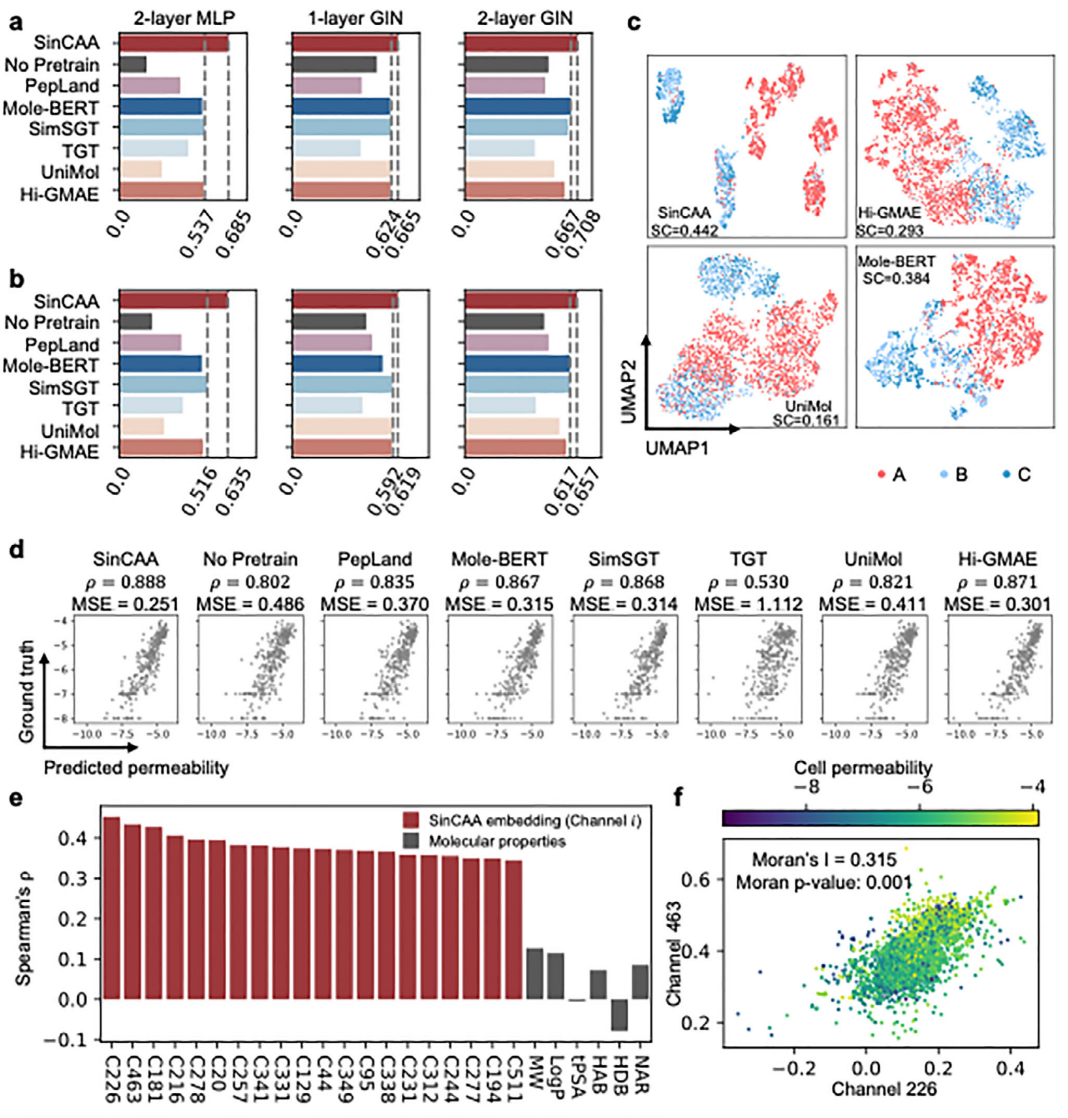
Results on predicting peptide selectivity and cell‐penetration ability. (a, b) Weighted F1 (a) and macro‐F1 score (b) of SinCAA and baseline methods for predicting NRAS/KRAS binding selectivity. Peptides were classified into three classes based on fold differences in binding affinity: Class A (≥tenfold, highest selectivity), Class B (≥threefold), and Class C (<threefold). SinCAA achieves higher F1 scores than baseline methods across both metrics. (c) UMAP visualization of peptide embeddings generated by different models, colored by selectivity class. SinCAA embeddings clearly separate Class A peptides from Classes B and C, demonstrating strong inherent discriminative power. The silhouette score (SC) calculated using the true class labels is 0.442. (d) Spearman's ρ and mean squared error (MSE) for predicting peptide cell‐penetrating ability. Using a two‐layer GIN encoder, SinCAA achieves the best performance among compared methods and lower MSE than a previously reported cell‐penetrating peptide prediction model. (e). Comparison of correlations between SinCAA embedding channels and experimental cell‐penetration measurements with correlations obtained from conventional molecular descriptors, including molecular weight (MW), LogP, topological polar surface area (tPSA), hydrogen bond acceptors (HBA), hydrogen bond donors (HBD), and number of aromatic rings (NAR). (f). Representative SinCAA embedding channels (channels 226 and 463) associated with cell‐penetration activity. The combined embedding representation achieves a Moran's I value of 0.315 with respect to experimental cell‐penetration scores, indicating positive spatial autocorrelation.

Modeling and optimizing selectivity is inherently more challenging than predicting binding affinity, as it requires capturing subtle differences in binding behaviors across homologous proteins. Here, we focused on selectivity between NRAS and KRAS, which share high sequence similarity yet exhibit differential binding to specific peptides. Selectivity was categorized into three classes (A, B, and C) based on fold differences in binding affinity: Class A represents the highest selectivity (≥tenfold), followed by B (≥threefold), and C (<threefold). Classification results are shown in Figure 3a,b and Figure S10. Using a two‐layer GIN as the downstream predictor, SinCAA embeddings achieved substantially higher F1 scores (macro, micro, and weighted) than all baseline methods. Furthermore, Figure 3c and Figure S11 show UMAP projections of peptide embeddings learned by different pretraining models, colored by selectivity class. Notably, SinCAA embeddings separated Class A peptides from lower‐selectivity peptides. These results suggest that SinCAA captures molecular features associated with selective binding, providing informative representations for peptide selectivity prediction in data‐limited settings.

We next evaluated SinCAA's ability to predict cell penetration using the CycPeptMPDB dataset [40]. Results in Figure 3d and Figure S12 show that, across simple models, including MLP, one‐layer GIN, and two‐layer GIN, SinCAA consistently outperformed other embedding methods. Notably, with a 2‐layer GIN, SinCAA achieved an MSE of 0.251, slightly outperforming the state‐of‐the‐art CPP model [18] (MSE was 0.253), despite the latter relying on hand‐engineered features and a specialized architecture tailored for cell‐penetration prediction. This highlights SinCAA's ability to learn transferable molecular representations from ncAA pretraining that remain effective for peptide‐level property prediction.

We also investigated whether SinCAA embeddings enable zero‐shot estimation of cell‐penetration ability, which, unlike binding affinity, is less dependent on the peptide's 3D structure. As shown in Figure 3e,f certain embedding channels exhibit higher correlations with experimental scores than conventional molecular descriptors associated with cell penetration, such as molecular weight, LogP, tPSA, HBA, HBD, and the number of aromatic rings. Combining the top two correlated channels yields a Moran's I of 0.315 relative to the experimental scores, suggesting that SinCAA embeddings encode information associated with cell‐penetration ability. We further compared the correlations of the top 20 embedding dimensions with those obtained from baseline methods using a one‐sided Mann–Whitney U test (Figure S13a). The top embedding channels of SinCAA exhibit significantly stronger correlations with the experimental scores than those of most baseline methods. The only exception is Hi‐GMAE, whose performance is comparable to SinCAA (p = 0.13).

Cell‐penetration prediction errors stratified by the number of ncAAs unseen during pretraining are shown in Figure S13b. Consistent with observations from Kd and IC50 prediction, these results provide no indication that downstream performance is driven by direct exposure to the same ncAA identities during pretraining.

To further evaluate the applicability of SinCAA beyond ribosomal peptides, we evaluated its performance on non‐ribosomal peptides (NRPs), a major class of therapeutically important molecules. Specifically, we focused on Arylomycin, which was initially identified as a narrow‐spectrum antibiotic; subsequent amino acid substitutions led to derivatives with broad‐spectrum antibacterial activity [41]. We searched ChEMBL [42] for arylomycin‐related studies and retained datasets with high‐quality experimental measurements and sufficient numbers of screened compounds. This resulted in two studies [43, 44], containing 68 and 31 molecules, respectively. Each study included three distinct antibacterial assays, corresponding to different measurements of inhibition against *Escherichia coli* strains. We evaluated the predictive performance of different embeddings using fivefold cross‐validation with random splits, using Ridge regression or Bayesian Ridge regression. The root mean squared error values are reported in Figure S14. Under these limited‐data settings, SinCAA achieved top‐1 performance across all six assays when using Ridge regression, and ranked first in five out of six assays when using Bayesian Ridge regression. Notably, antibacterial activity against *E. coli* is mechanistically complex and may depend on multiple factors, including bacterial penetration and effective binding to the target protein. These results demonstrate that even for such multifactorial properties and under severe data scarcity, SinCAA embeddings remain highly informative and practically useful.

In summary, these results demonstrate that SinCAA effectively models a broad range of peptide and peptide‐like molecular properties and consistently outperforms baseline methods. To illustrate a practical application of SinCAA for peptide optimization, we selected five low‐permeability peptides from the test set and performed in silico residue substitution screening. Candidate substitutions were generated based on conformational similarity and ranked using the same two‐layer GIN permeability predictor used in Figure 3 (Figure S15). These examples are intended to demonstrate a potential workflow enabled by SinCAA rather than provide experimentally validated design recommendations.

### 2.4 Predicting General Protein Peptide Binding With SinCAA

The downstream tasks described above primarily involved peptide datasets with relatively similar molecular scaffolds and a high prevalence of ncAAs. To further evaluate the versatility of SinCAA, we investigated its applicability to general peptides containing ncAAs for predicting protein–peptide interactions.

As existing peptide binding datasets poorly represent ncAAs, we first curated a comprehensive protein–peptide binding dataset from the Protein Data Bank. To enable protein–peptide interaction prediction, we replaced the peptide embedding module in PepNN  [22] with pretrained SinCAA embeddings and added losses to supervise peptide binding sites. PepNN is a model originally designed to predict peptide binding sites on proteins based on protein structures and peptide sequences. For simplicity, we refer to this modified model as SinCAA. We benchmarked SinCAA against two state‐of‐the‐art protein–peptide interaction models, CAMP [21] and PepGPL [23]. Notably, CAMP and PepGPL encode all ncAAs simply as a placeholder and do not account for cyclic peptide structures, limiting their ability to model chemically diverse peptides.

**FIGURE 4:**
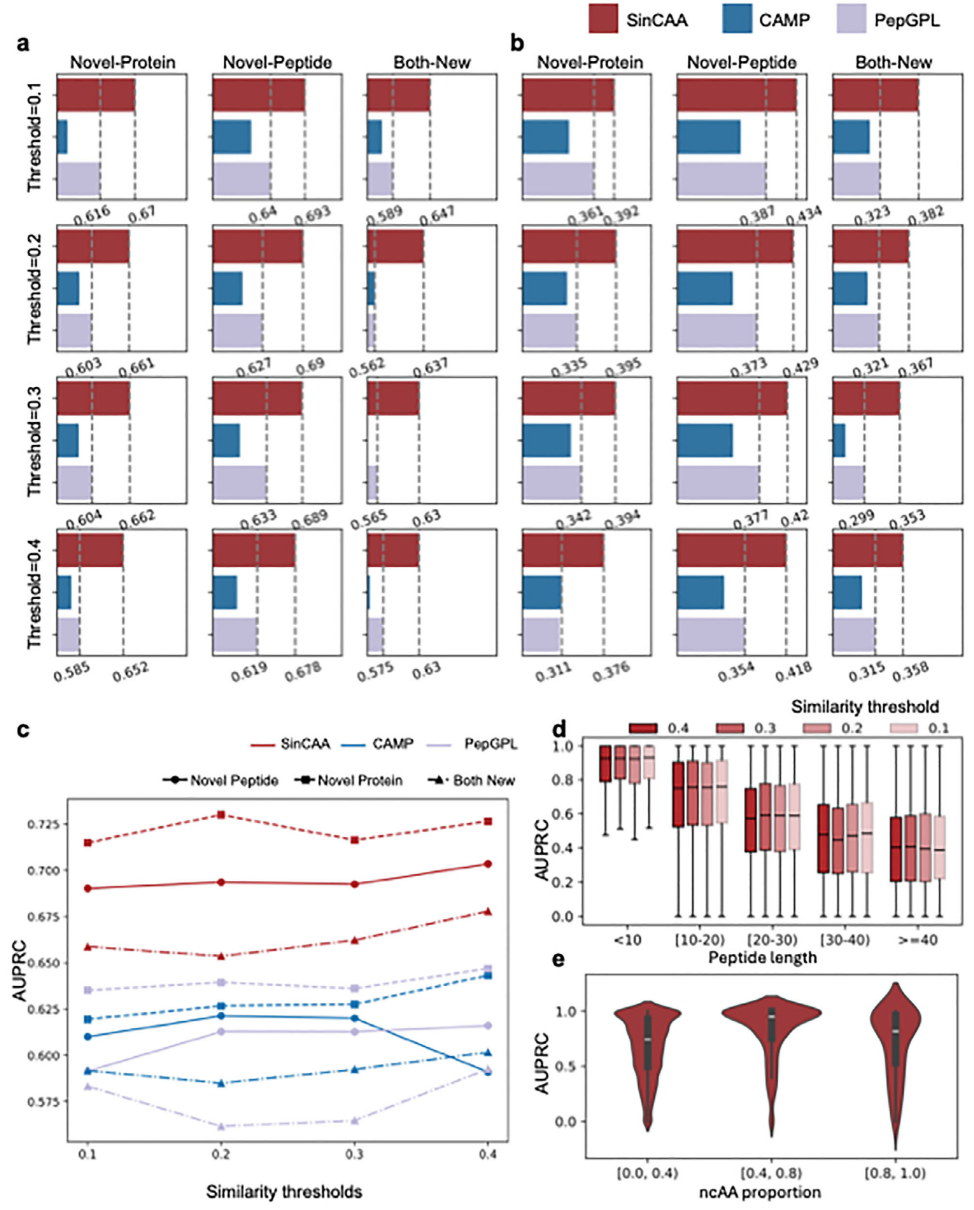
Prediction of general protein–peptide binding. (a, b) Area under the precision–recall curve (AUPRC; a) and Matthews correlation coefficient (MCC) at a prediction threshold of 0.5 (b) for different methods in predicting peptide binding sites under different sequence similarity thresholds and data‐splitting strategies. In these experiments, the peptide encoding module of PepNN was replaced with molecular embeddings generated by SinCAA or Hi‐GMAE. For simplicity, models are labeled according to the embedding method used. (c) Comparison of methods for predicting binding sites of ncAA‐containing peptides under different similarity thresholds and splitting strategies. (d) AUPRC scores stratified by peptide length. (e) AUPRC scores stratified by ncAA proportion.

Following protocols in the literature [21, 22, 23], we evaluated model performance under three data‐splitting strategies: novel proteins, novel peptides, and both‐new proteins and peptides. For each strategy, we applied varying similarity thresholds to define the dissimilarity between training and testing sets, where higher thresholds correspond to lower similarity between the two sets. Figure 4a,b as well as Figure S16, show the performance for predicting peptide binding sites across all peptides in the dataset, including both linear peptides composed of only canonical amino acids and cyclic peptides containing ncAAs. Across all splitting strategies and similarity thresholds, models using pretrained peptide embeddings consistently outperformed both CAMP and PepGPL. Furthermore, to demonstrate the superiority of SinCAA over small molecule pretraining methods, Figure S17 shows that models with SinCAA embeddings consistently outperformed those using Hi‐GMAE embeddings, albeit modestly, possibly because most amino acids present in peptides within the PDB dataset are canonical residues. This result highlights SinCAA's effectiveness in modeling general peptides. The performance of different methods in predicting protein binding sites is shown in Figure S18. No substantial improvement was observed when using pretrained peptide embeddings relative to the original PepNN, indicating that peptide representations contribute less on this task.

Next, we isolated peptides containing ncAAs from the dataset to directly compare the performance of different methods in predicting binding sites of ncAA‐containing peptides. As shown in Figure 4c and Figure S19, SinCAA consistently outperformed state‐of‐the‐art peptide binding site prediction methods across all settings, with almost no performance decrease as the dissimilarity between training and test datasets increased. This highlights the robustness of SinCAA embeddings across diverse scenarios and further demonstrates that existing peptide binding site prediction methods are less effective for modeling peptides containing ncAAs.

Finally, we explored the performance of SinCAA across peptides of varying lengths and ncAA proportions (Figure 4d,e). As peptide length increases, the AUPRC score of SinCAA decreases, likely due to the reduced proportion of binding sites in longer peptides, yet it remains stable across different similarity thresholds. As the proportion of ncAAs increases, the AUPRC score remains largely unchanged, indicating the robustness of SinCAA embeddings in handling chemically diverse peptides.

Taken together, these results highlight that SinCAA generalizes effectively to diverse peptide structures and consistently outperforms existing methods in protein–peptide binding prediction tasks, despite being pretrained exclusively on individual ncAAs rather than peptide sequences or structures.

### 2.5 SinCAA Exhibits Good Generalizability From Public to Pharmaceutical Peptide Data

The final question we sought to address, arguably the most challenging in AI‐assisted therapeutic peptide development, is whether a model trained solely on public data can generalize effectively to in‐house datasets. Compared with public peptide–protein datasets, pharmaceutical peptide data are often composed of chemically diverse ncAAs and unique cyclic topologies that cannot be adequately represented as simple amino acid sequences. Consequently, the PepNN‐based models described in the previous section, which represent peptides as residue sequences, are not directly applicable to this setting.

To overcome this limitation, we adapted the PepNN framework by modifying the peptide representation from the residue level to the atom level. Specifically, we removed the averaging step that aggregates atom‐level features into residue‐level embeddings, thereby allowing the model to capture chemical features at the atomic scale. The modified model was trained using fivefold cross‐validation under a novel‐peptide split with a similarity threshold of 0.3, and evaluated on its ability to predict the Kd of peptides in the KRAS binding dataset described earlier. To avoid potential data leakage, protein complexes containing peptides highly similar to the test set (PDB IDs 7YV1, 8JJS, and 7YUZ) were excluded from training, as they were proposed in studies [33, 45] closely related to the KRAS dataset.

It is important to emphasize two major gaps between the training and evaluation tasks. First, the peptide distributions differ substantially: training peptides bear little resemblance to the evaluation peptides. Second, the training objective was binding site prediction, defined by spatial proximity of protein–peptide contacts, whereas the evaluation task was binding affinity prediction, which depends on more intricate biophysical interactions. For evaluation, we leveraged the PepNN framework to test KRAS structures from four different PDB entries: the three complexes containing peptides similar to the test set (7YV1, 8JJS, 7YUZ) derived from related KRAS studies, and an unrelated KRAS structure (7ROV) from an independent study [46], which includes the G12D mutation and a peptide ligand.

Prediction scores were derived from the two‐dimensional protein–peptide site prediction matrix, in which rows correspond to peptide atoms and columns correspond to protein residues. For each peptide, we first averaged the predicted scores across peptide atoms to obtain a single score per protein residue, and then took the maximum of these per‐residue scores as the peptide's overall prediction score, corresponding to its predicted interaction with the most important protein binding site. To benchmark SinCAA embeddings against small‐molecule pretraining approaches, we also trained a model using Hi‐GMAE embeddings under the same scheme.

**FIGURE 5:**
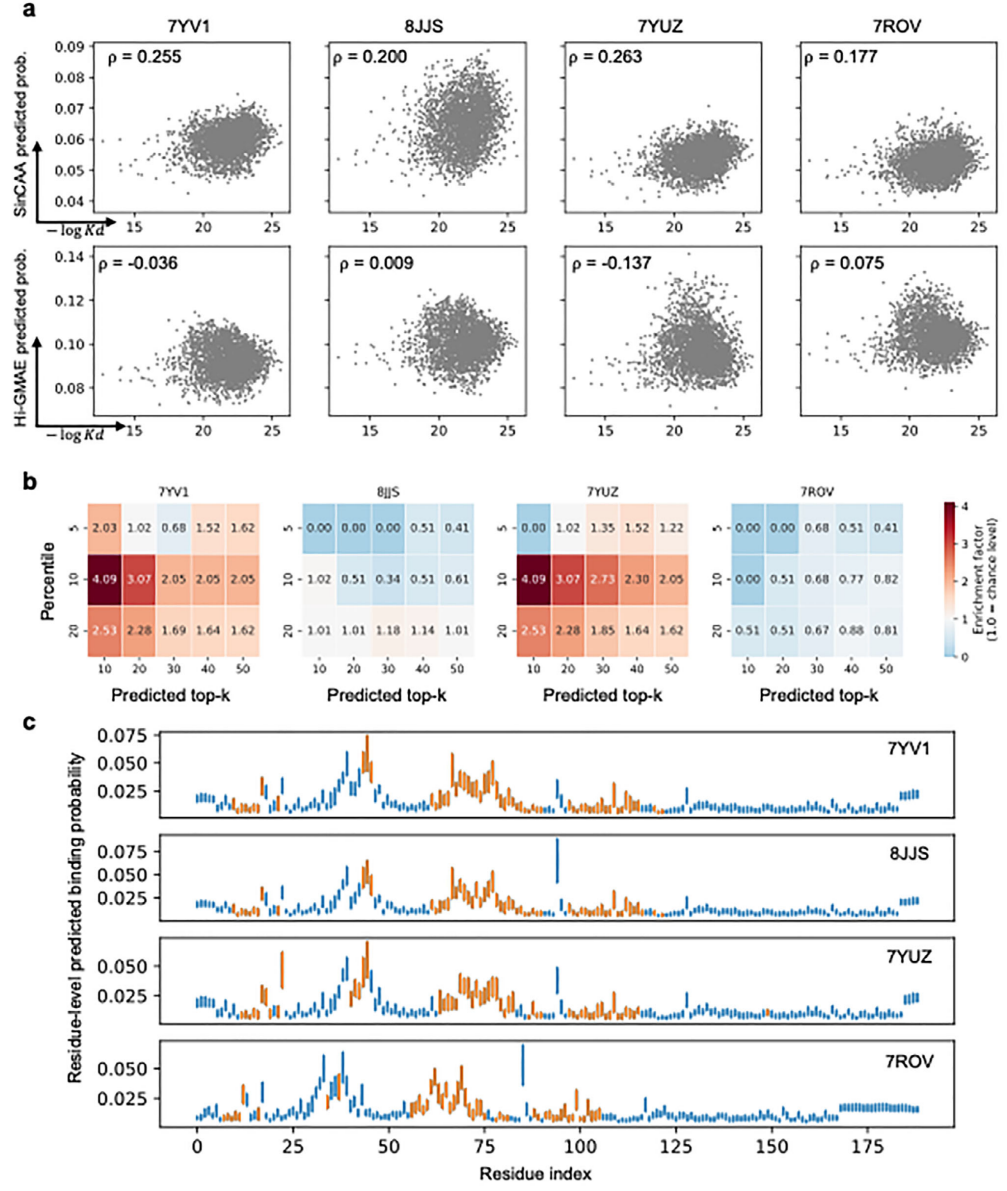
Generalization ability of SinCAA from public datasets to an independent peptide dataset. (a) Correlation between experimental −log(Kd) values and predicted scores generated by different models. (b) Enrichment of high‐affinity peptide binders among top‐ranked SinCAA predictions for four KRAS structures. Enrichment factors (EFs) were calculated for 7YV1, 7YUZ, 8JJS, and 7ROV using different affinity thresholds defining active peptides (top 5%, 10%, or 20% by experimental binding affinity; rows) and different numbers of top‐ranked predictions (Top‐k = 10, 20, 30, 40, and 50; columns). EF > 1 indicates enrichment relative to random selection. (c) Average predicted scores across protein residue indices, with experimentally defined pocket residues (within 10 Å of the peptide) highlighted. For 7YV1 and 7YUZ, the top‐1 predicted residue overlaps with the known peptide‐binding pocket, whereas this overlap is absent for 7ROV and 8JJS.

As shown in Figure 5a, models using SinCAA embeddings achieved modest but consistent correlations between predicted scores and experimental −log(Kd) values across all four KRAS structures and substantially outperformed models using Hi‐GMAE embeddings. Furthermore, Figure 5b demonstrates the enrichment of high‐affinity peptide binders among top‐ranked predictions. For 7YV1 and 7YUZ, the top‐10 predicted peptides achieved enrichment factors of 4.09 and 2.53 for identifying peptides within the top 10% and top 20% of experimental binding affinity, respectively, compared with random selection. These results suggest that SinCAA‐based predictions can effectively prioritize promising candidates and potentially reduce experimental screening efforts. For structures 8JJS and 7ROV, the predicted scores did not achieve significant enrichment over random selection. Further analysis suggests that this limitation mainly arises from inaccurate binding‐pocket localization: as shown in Figure 5c for 7YV1 and 7YUZ, the protein regions with the highest average predicted binding probabilities overlap with the peptide‐binding pockets, whereas this was not observed for 8JJS and 7ROV. To determine whether this reflects a failure of peptide‐level ranking or of protein‐side localization, we performed a post‐hoc diagnostic analysis in which the top‐1 predicted site outside the experimentally known binding pocket was excluded before recomputing enrichment. This substantially improved the enrichment factor for both 8JJS and 7ROV (Figure S20), indicating that the underlying peptide‐ranking signal remains informative once the correct binding site is identified. These results indicate that the major limitation in these cases likely originates from protein‐side binding‐site prediction rather than peptide representation, as the underlying model architecture was adapted from PepNN with the peptide embedding component replaced by SinCAA.

Together, these results demonstrate that SinCAA representations transfer effectively from large‐scale public ncAA datasets to proprietary pharmaceutical peptide optimization tasks when reliable binding‐site localization is available. Despite substantial differences in peptide chemistry, data distributions, and training objectives, SinCAA consistently outperformed small‐molecule pretrained representations, enabling improved prioritization of chemically diverse macrocyclic peptide binders.

## 3 Discussion

In this work, we proposed SinCAA, a similarity‐enhanced pretraining framework specifically designed for therapeutic peptides containing ncAAs. Although pretrained exclusively on ncAAs rather than peptide data, SinCAA learned transferable molecular representations that effectively capture peptide‐relevant chemical features. These representations supported accurate prediction across diverse downstream tasks, including binding affinity, binding selectivity, and cell penetration. Notably, SinCAA exhibited strong generalization, including zero‐shot performance, highlighting its potential for accelerating AI‐assisted therapeutic peptide discovery.

We systematically benchmarked SinCAA across diverse tasks, including protein–peptide interaction site prediction and property‐specific tasks. SinCAA enables identification of peptides with high target affinity, critical for therapeutic efficacy, and predicts binding selectivity, helping minimize off‐target effects and improve drug safety. Cell‐penetrating ability prediction guides the design of peptides with better intracellular delivery and bioavailability, while interaction site prediction supports rational optimization of peptide–protein interfaces. Across all tasks, SinCAA consistently outperformed baseline methods, with the advantage most pronounced under low‐data and zero‐shot conditions, demonstrating its ability to leverage chemically diverse ncAA information to accurately model peptide properties and accelerate candidate screening in therapeutic peptide discovery.

Despite these encouraging results, several limitations remain. First, although SinCAA's predictions were extensively benchmarked against experimentally measured properties across multiple independent datasets, our study focused on retrospective evaluation of predictive tasks using existing experimental datasets; we did not assess the utility of SinCAA embeddings for generative modeling, such as de novo peptide sequence design, nor did we prospectively apply the framework to screen newly designed peptides followed by wet‐lab experimental validation. Extending SinCAA to generative objectives and validating its use for novel peptide design through experimental screening represent natural and impactful directions for future work. Second, our conformational similarity metric was integrated through contrastive learning, which emphasizes relative similarity ranking rather than absolute similarity values. While this design aligns with peptide engineering practices, where relative substitutability is often more relevant, it may also limit performance. Future extensions could explore hybrid strategies that more directly incorporate similarity scores, potentially further improving embeddings. Finally, although our analyses suggest that SinCAA captures meaningful biochemical features, the mechanisms behind its strong generalization remain underexplored. Deeper interpretability studies could yield insights into the chemical and structural knowledge embedded within the model and guide the development of even more powerful architectures.

Overall, our findings demonstrate that SinCAA serves as a robust foundation model for therapeutic peptide design, bridging ncAA representations with peptide‐level properties. By providing accurate, generalizable embeddings, SinCAA enables accelerated virtual screening and prioritization of ncAA‐containing peptide candidates. In the absence of task‐specific training data, SinCAA embeddings can support preliminary candidate prioritization, while the conformational similarity metric provides an efficient strategy for identifying promising residue substitutions during peptide optimization. When limited experimental data are available, downstream models trained on SinCAA embeddings can achieve substantially improved predictive performance. With further prospective validation and extension to generative modeling, SinCAA could become a useful component of AI‐driven peptide discovery pipelines, facilitating the efficient identification and optimization of therapeutically relevant peptide candidates.

## 4 Method

### 4.1 Non‐Canonical Amino Acid Dataset

SMILES representations of ncAAs were retrieved from PubChem (https://pubchem.ncbi.nlm.nih.gov/) using the keyword *“amino acid”* on March 13, 2024. The dataset was curated by removing entries that could not be parsed by RDKit, as well as those lacking the characteristic amino acid substructure (identified using the SMARTS pattern [N!H0]‐[C:1]‐[C:2](= [O:3])‐[OH]). Molecules containing more than 30 atoms were also excluded to limit the dataset to amino acid‐sized molecules. After filtering, a total of 387 482 ncAA SMILES strings were retained. The dataset was randomly split into training and validation sets at a 8:2 ratio, yielding 309 986 molecules for training and 77 496 for validation. No additional standardization of protonation states, tautomeric forms, or stereochemistry was performed; molecules were processed as deposited in PubChem. This represents a limitation of the current data‐processing pipeline, and systematic standardization across the full dataset may further improve the conformational similarity metric.

### 4.2 ZINC15 Dataset (10 M)

A subset of 10 million molecular structures was obtained from the ZINC15 database in SMILES format using the DeepChem API. This dataset was also randomly divided into training and validation sets in an 8:2 ratio, with 8 million molecules used for training and 2 million for validation. This dataset served as an auxiliary pretraining dataset to improve the model's ability to learn general chemical features.

### 4.3 PDB Dataset

Protein structures were downloaded from the RCSB PDB website (https://www.rcsb.org/) on October 23, 2023. We followed the pipeline of OpenFold [47] to parse mmCIF files and retained chains of length 2–50 residues, which were treated as peptides. For each mmCIF file, contact areas between peptides and proteins within the same assembly were computed.

Peptides were considered to bind proteins if the contact area exceeded 200 Å^2^, a threshold derived from the distribution of contact areas and more stringent than those used in previous studies [48]. Only assemblies containing at least one peptide–protein interaction were retained. Subsequently, peptide sequences from the remaining files were extracted, and entries with broken peptide chains were excluded, resulting in a final set of 12 131 protein–peptide interaction instances. Notably, unlike existing datasets where each peptide interacts with a single protein, our dataset allows a peptide to interact with multiple proteins, with each interaction treated as a distinct data point. For each interaction, the minimum interatomic distance between peptide and protein atoms was computed. To define the binding site, we used the 0.98 quantile of all minimum distances to mitigate long‐tail effects, yielding a threshold of 3 Å. Binding sites on both peptides and proteins were defined accordingly. Distances were computed based on all atoms rather than only Cα atoms, as side‐chain atoms play a crucial role in non‐covalent interactions during peptide–protein binding.

To split the data for model evaluation, we followed a clustering‐based strategy commonly used in prior studies [21, 22, 23, 49]. First, pairwise sequence similarities were computed using the Smith–Waterman algorithm for proteins or peptides. These similarity scores were then used for hierarchical clustering with varying similarity thresholds. For the novel protein and novel peptide settings, clustering was conducted separately on the protein and peptide sequences, respectively. The resulting clusters were then divided into five folds. In each cross‐validation iteration, four folds were used for training and one fold for testing. The training portion was further randomly split into training and validation sets at a 9:1 ratio. For the both‐new setting, clustering was performed independently on protein and peptide sequences, each split into three folds. Their Cartesian product resulted in nine folds. In each iteration, eight folds were used for training and one fold for testing. As above, the training data were further randomly split into training and validation sets at a 9:1 ratio.

### 4.4 CycPeptMPDB Dataset

The CycPeptMPDB dataset [40] was downloaded from the official website (http://cycpeptmpdb.com/download/). For model training and evaluation, we followed the protocol described in the literature [18] and adopted the same data split to ensure consistency. Specifically, we used experimentally measured permeability values obtained from the PAMPA assay. When multiple measurements were reported for the same peptide, the value from the most recent publication was retained. The logarithmic permeability values were clipped to the range [−8, −4] and used as regression labels. A total of 6889 peptide sequences were included. Following the original split in 18, 344 peptides were used as the test set, while the remaining samples were used for training. The remaining peptides were further divided into training and validation subsets using three different random seeds. For each split, three independently initialized models were trained, resulting in a total of nine models. Test‐set predictions were averaged across all nine models, and the corresponding performance metrics were reported.

### 4.5 KRAS Peptide Binding Dataset

The KRAS peptide binding dataset used in Figures 2 and 3 was extracted from the publicly available patent US20240158446A1 [39], which reports experimental measurements including Kd, IC50, and NRAS/KRAS selectivity for a total of 2761 peptides. These peptides range from 10 to 12 amino acids in length and include both monocyclic and bicyclic structures. Given the high sequence similarity among these peptides, we computed pairwise similarities using MACCS keys fingerprints. Among several fingerprinting methods tested, MACCS keys provided the greatest distinction between training and test sets when used for clustering, and was thus selected for data splitting. Based on this similarity matrix, peptides were partitioned into two clusters using KMeans clustering, with each peptide represented as a similarity vector over the entire dataset. This resulted in 1858 peptides for model training and 903 peptides for testing. The training set was further split into training and validation subsets at an 8:2 ratio. To mitigate the impact of random seed variability, each method was run three times with different seeds, and average performance metrics were reported.

To assess the zero‐shot capability of different similarity metrics, we analyzed all 14 peptide groups within the 2761‐entry dataset that differ by a single‐position mutation and contain more than 10 peptides per group. Among these, two groups involve substitutions at terminal positions rather than complete amino acids. Since conformational similarity can only be computed for full amino acids, our evaluation focuses on the remaining 12 peptide groups.

The KRAS binding data used in Figure S1 and Table S1 were sourced from Table S2 of Reference 9, which reports a set of cyclic peptides differing at position 1 while keeping the rest of the sequence unchanged. The starting amino acid at position 1 was MeAla.

For the structural analysis shown in Figure 5, PDB entries 7YV1, 8JJS, and 7YUZ correspond to peptides similar to those in the KRAS dataset used here [33, 45], though the peptides themselves are not included in the dataset. PDB entry 7ROV, from Reference 46, corresponds to a KRAS peptide binder unrelated to the KRAS binding dataset used for evaluation.

### 4.6 Arylomycin Dataset

ChEMBL was searched using the structures of Arylomycin A2, Arylomycin C16, and G0775 as queries with a similarity threshold of 0.4 to maximize coverage of the arylomycin family. Assays measuring antibacterial activity against Escherichia coli were retained, and studies containing fewer than 20 compounds were excluded. This yielded three studies: CHEMBL1811847, CHEMBL5518248, and CHEMBL5370681. CHEMBL1811847 was excluded because all activity measurements were reported at a resolution of 1 µg/mL. The remaining two studies comprise six independent assay datasets, with no overlap between assays. All activity measurements were transformed to negative logarithmic values before model training.

### 4.7 Calculation of Conformational Similarity

Conformers of ncAAs were generated using the ETKDG algorithm implemented in RDKit, without subsequent energy minimization, conformer deduplication, energy‐based filtering, or energy‐based reweighting. All generated conformers for each ncAA were treated with equal weight. Partial charges were calculated using RDKit's implementation of Gasteiger–Marsili partial charges, selected for computational efficiency at the scale required for this study.

To compute conformational similarity between two ncAAs, we first define a similarity metric between their conformations. Because the coordinates of a molecular conformation can vary with the choice of reference frame, directly comparing atomic positions is not meaningful. Since all ncAAs share a common amino acid substructure, we standardize each conformation by establishing a canonical coordinate system. Specifically, the α‐carbon is placed at the origin, the nitrogen of the amino group is aligned along the negative x‐axis, and the xy‐plane is defined using the nitrogen and carboxyl carbon atoms. This standardization ensures that conformations of different ncAAs are represented in a consistent frame, allowing their spatial features to be directly compared.

Since ncAAs differ in structure, directly computing root‐mean‐square deviation between their conformations is not reasonable. To enable spatial comparison across different ncAAs, we discretize the 3D space into a grid with a predefined grid size *h*. For each grid cell, we record atom‐type–specific information, including element types and partial charges. If multiple atoms fall into the same cell, atoms of the same element are merged by summing their charges, while atoms of different elements are stored as separate entries. This process yields a set representation of a conformation $c_{a}^{\left(i\right)}$, denoted as $R\;\left(c_{a}^{\left(i\right)}\right)=\left\{r\right\}$, where each *r* corresponds to a non‐empty grid cell containing element‐specific charge information. To measure the similarity between two conformations $c_{a}^{\left(i\right)}$ and $c_{b}^{\left(i\right)}$, we define a charge‐weighted Jaccard similarity:

$$\begin{array}{cc} & S\left(R\left(c_{a}^{\left(i\right)}\right),\;R\left(c_{b}^{\left(j\right)}\right)\right)\\ & \;=\frac{\sum_{r\in R\left(c_{a}^{\left(i\right)}\right)\cap R\left(c_{b}^{\left(j\right)}\right)}S_{cell}\left(r\right)}{\left|R\left(c_{a}^{\left(i\right)}\right)\right|+\left|R\left(c_{b}^{\left(j\right)}\right)\right|-\sum_{r\in R\left(c_{a}^{\left(i\right)}\right)\cap R\left(c_{b}^{\left(j\right)}\right)}S_{cell}\left(r\right)} \end{array}$$

Here, *S_cell_* (*r*) is the similarity score for a shared grid cell *r*, defined as:

$$S_{cell}\;\left(r\right)=\left\{\begin{array}{c} \frac{1}{\left|E_{r}\right|}\sum_{e\in E_{r}}\max\left(1-\left|q_{a}^{\left(i\right)}\left(r,e\right)-q_{b}^{\left(j\right)}\left(r,e\right)\right|,\;0\right),\;\left|E_{r}\right|>0\\ 0,\;otherwise \end{array}\right.$$

where, *E_r_* is the set of shared atom elements in grid cell *r* between conformation $c_{a}^{\left(i\right)}$ and $c_{b}^{\left(j\right)}$, and $q_{a}^{\left(i\right)}\left(r,e\right)$ and $q_{b}^{\left(j\right)}\left(r,e\right)$ denote the charges of element *e* in cell *r* for each conformation.

Building on the similarity between individual conformations, we define the overall similarity between two ncAAs. Since each ncAA can adopt multiple stable conformations, we treat its structure as a distribution rather than a single fixed conformation. To approximate the similarity between two such distributions, we sample *n* conformations per ncAA (*n*  =  20 in our experiments) and compute all pairwise similarities between conformations of the two ncAAs. We then apply the Hungarian algorithm to align conformations across the two ncAAs, deriving a bijection π(*i*) that assigns each *i*th conformation of molecule *a* to its optimal match in molecule *b*, thereby maximizing total similarity. The average similarity over these aligned pairs is taken as the conformational similarity *S_conf_* (*a*,*b*) between the two ncAAs. This approach provides an efficient approximation of the Earth Mover's Distance between their conformation distributions. As shown in Figure S1, the resulting conformational similarity is robust to the choice of both *h* and *n*.

To construct training pairs for contrastive learning, we identify, for each ncAA, its 20 most similar amino acids from the curated ncAA dataset. To accelerate the search, the candidate pool is restricted to amino acids whose atom count differs by no more than two from the query ncAA. The search is performed hierarchically: first, similarities are computed at a coarse grid resolution of 2.0 Å to retrieve the top 300 candidates; next, these candidates are re‐evaluated at a finer resolution of 1.0 Å to select the top 60; finally, the top 20 most similar amino acids are chosen from these 60 using the finest grid resolution of 0.5 Å. These top 20 ncAA pairs are used as positive pairs in contrastive learning. For each anchor ncAA, negative samples are implicitly defined as all other samples in the same mini‐batch that are not among its positive pairs. This in‐batch negative sampling strategy is widely adopted in contrastive learning to enhance both efficiency and the diversity of negative examples. Unless otherwise specified, conformational similarity in this work was computed with *h*  =  0.5 and *n*  =  20. Using the three‐stage hierarchical search strategy and caching of precomputed conformations, computing pairwise conformational similarity across all ncAAs required approximately 50 000 CPU‐hours in total.

### 4.8 Pretraining of SinCAA

As illustrated in Figure 1a, the pretraining of SinCAA consists of two tasks: masked node recovery and contrastive learning. For the masked node recovery task, following established approaches in graph pretraining [26, 27, 28, 50], 50% of atoms in each molecular graph are randomly masked, and the model is trained to reconstruct the masked atom types using a cross‐entropy loss. For the contrastive learning task, the loss is defined as:

$$\begin{array}{cc} & \;l_{contrast}\\ & \;=E\left[-\log\frac{\exp\left(-{\left|f\left(x^{+}\right)-f\left(x\right)\right|}_{2}\right)}{\exp\left(-{\left|f\left(x^{+}\right)-f\left(x\right)\right|}_{2}\right)+\sum_{i}\exp\left(-{\left|f\left(x_{i}^{-}\right)-f\left(x\right)\right|}_{2}\right)}\right], \end{array}$$

where *f*(*x*) denotes the embedding of an ncAA *x* and *x* ^+^ and *x* ^−^ represent the positive and negative pairs of *x*, respectively. We adopt an in‐batch negative strategy, treating all samples other than the positive as easy negatives within each batch. In‐batch negatives, however, are largely random and thus easy to distinguish from the positive, limiting the model's ability to learn fine‐grained relative similarity. To address this, for each amino acid, we randomly sampled two ncAAs from its top‐20 neighbors ranked by conformational similarity and assigned labels based on their relative similarity: the more similar candidate served as the positive sample x^+^, and the less similar one as a hard negative. The architecture of SinCAA is based on the GPS Transformer [35], with a 2‐layer GIN [36] encoder to capture local structural information. We employ a 4‐layer GPS Transformer with a hidden size of 512. A one‐layer GIN decoder is used for node recovery, consistent with standard graph pretraining protocols [26, 27, 28]. The total number of parameters in SinCAA is 13.6 million.

Given the limited number of ncAAs available for training, we augment the data with 10 million molecules from the ZINC15 database, which are used exclusively for the node recovery task. Each pretraining epoch consists of 3125 steps with a batch size of 640, considering the size of the ZINC15 dataset. The model was trained for 20 epochs using four NVIDIA A100 GPUs with a learning rate of 0.001. The same training setup is applied across all ablation studies.

### 4.9 Training and Evaluation of Models on Downstream Tasks

For the experiments shown in Figures 2 and 3, we evaluated three different downstream architectures: a two‐layer multilayer perceptron (MLP), a one‐layer GIN with a hidden size of 256, and a two‐layer GIN with the same hidden size. Each model was trained with an initial learning rate of 0.001 for 300 epochs. The batch size was set to 32 for the KRAS peptide binding dataset, with an exponential learning rate decay of 0.99, and 128 for the cell penetration dataset without learning rate decay.

For the experiments in Figure 4, we modified the PepNN architecture by replacing its trainable peptide embeddings with pretrained embeddings from SinCAA and Hi‐GMAE. The self‐attention mechanism in the peptide module was removed while keeping all other components unchanged. We also introduced losses for peptide binding site prediction and protein–peptide interaction site prediction, using binary cross‐entropy for each position. Following the original PepNN training protocol, models were trained for 20 epochs with a batch size of 1. The PepNN feature extraction module was further extended to support multiple protein targets. As CAMP and PepGPL do not support multi‐protein input during evaluation, for a fair comparison, multi‐protein data were decomposed into multiple peptide–single‐protein pairs for training. During evaluation, the maximum predicted value across all associated protein sequences was used as the final prediction for each peptide.

For the experiments in Figure 5, the peptides in the KRAS binding dataset could not be decomposed into sequences, so previous models could not be applied directly. We trained a new model by replacing residue‐level peptide representations with atom‐level representations, while keeping all other components unchanged. Training was performed using fivefold cross‐validation with a similarity threshold of 0.3 for the novel‐peptide split, after removing entries with peptide structures similar to those in the KRAS dataset (7YV1, 8JJS, and 7YUZ). The prediction results in Figure 5 were obtained by averaging outputs across the five models.

### 4.10 Baseline Methods

SinCAA was compared with representative molecular and peptide pretraining approaches, including UniMol [51], Hi‐GMAE [27], Mole‐BERT [52], TGT [53], SimSGT [54] and PepLand [24]. All baseline models were evaluated using publicly released implementations and pretrained checkpoints with default inference configurations unless otherwise specified.

1. **UniMol**: We employed the official UniMol implementation with standard pretrained weights and default hyperparameters to extract atom‐level embeddings from our datasets.
2. **Hi‐GMAE**: The hierarchical graph masked autoencoder baseline was implemented using the official codebase with released pretrained weights and default configurations. Atom‐level embeddings were extracted from the first coarse layer following the standard inference protocol.
3. **Mole‐BERT**: We utilized the pretrained Mole‐BERT model with default inference configurations as specified in the official repository. The BERT‐based molecular language model generated atom‐level embeddings through its standard tokenization and encoding pipeline.
4. **TGT**: We utilized TGT with its pretrained model checkpoint and default parameters to extract atom‐level embeddings from our datasets.
5. **SimSGT**: We employed the original SimSGT repository with default pretrained weights and standard configurations. The inference pipeline strictly adhered to the recommended settings to obtain atom‐level molecular embeddings.
6. **PepLand**: We utilized the pretrained PepLand model with its default inference settings as provided in the official repository to generate peptide representations for our datasets.

### 4.11 Dimension Reduction

The UMAP visualizations shown in Figure 3 and Figure S11 were generated using 40 neighbors, a minimum distance of 0.5, the Euclidean distance metric, and a random seed of 42.

### 4.12 Morgan Similarity

All Morgan similarity values reported in the manuscript were computed as Tanimoto similarity between Morgan fingerprints (radius = 2, 2048 bits; equivalent to ECFP4), generated with chirality flags enabled.

## Author Contributions

**Chencheng Xu** conceived the idea, designed the project, performed data cleaning, implemented the code, and drafted the manuscript. **Lesong Wei** contributed to data curation, executed baseline methods, and assisted in manuscript drafting. **Jianmin Wang** assisted in drafting the manuscript. **Yuanpeng Xiong**, **Ruochi Zhang**, and **Yu Wang** contributed to the curation of the KRAS binding peptide dataset. **Chao Zha** assisted with running baseline methods. **Qiangcheng Zeng** and **Xin Gao** supervised the project, contributed to manuscript drafting, and provided computational resources.

## Ethical Statement and Informed Consent

The authors have nothing to report

## Conflicts of Interest

The authors declare no conflicts of interest.

## Supporting information




**Supporting File**: advs77511‐sup‐0001‐SuppMat.docx.

## Data Availability

The data supporting the findings of this study are openly available in the SinCAA repository: https://github.com/Zoesgithub/SinCAA.git. Specifically, the repository provides the pretrained SinCAA model weights (https://github.com/Zoesgithub/SinCAA/tree/main/data/results/n1_weight0.1_innl2_both), the curated non‐canonical amino acid dataset (https://github.com/Zoesgithub/SinCAA/tree/main/data/AAList), and the processed PDB IDs for protein‐peptide binding (https://github.com/Zoesgithub/SinCAA/tree/main/experiments/1_ppb/raw_data/make_data). Two datasets used in this study are not included in the repository. The curated KRAS‐binding peptide dataset is available from the corresponding author upon request, while the Arylomycin dataset is publicly accessible through ChEMBL using the corresponding ChEMBL IDs. All other datasets used in this study, beyond those described above, are publicly available from their respective official repositories or the publications in which they were originally reported.
