# Supplementary material for: Similarity‐Enhanced Representation Learning of Non‐Canonical Amino Acids for Therapeutic Peptide Modeling
Source: Adv Sci (Weinh). 2026 Sep 1:e77511. Online ahead of print. doi: 10.1002/advs.77511 (PMC13534795; doi:10.1002/advs.77511)
Supplement: Supplementary file 1 [file ADVS-9999-e77511-s001.docx]

**Supplementary Figures**

**Supplementary Fig. 1: Spearman’s correlation of conformational similarity and Morgan similarity in predicting affinity changes after amino acid substitution.** The dataset includes substitutions at position 1 of a cyclic peptide. MeAla at this position results in the highest peptide binding affinity, and all similarities were calculated relative to MeAla. In principle, substitutions between more similar amino acids are expected to result in smaller changes in binding affinity. Conformational similarity was computed under different grid sizes $h$ and numbers of sampled conformations $n$, and the results indicate that its ranking is robust to these hyperparameters. Conformational similarity achieved a Spearman’s correlation of 0.83, compared with 0.6 for Morgan similarity.

**Supplementary Fig. 2: The conformational similarity metric distinguishes Cα-inverted (enantiomeric) ncAA pairs more effectively than Morgan fingerprint similarity.** Conformational similarity and Morgan fingerprint similarity were computed between each ncAA and (i) its Cα-inverted counterpart and (ii) the reference residue MeAla (the highest-affinity residue at this position). The conformational metric consistently assigns lower similarity to Cα-inverted pairs (mean ≈ 0.24, range 0.16–0.37) than to ncAA–MeAla pairs, whereas Morgan fingerprint similarity assigns higher similarity scores to Cα-inverted pairs.

**Supplementary Fig. 3: Overlap of d-patterns between ncAAs and peptides in the KRAS dataset (left) and the cell penetration dataset (right).** The overlap ratio between ncAAs and peptides increases proportionally with the number of appended ncAAs. To balance model performance and training cost, one or two appended ncAAs were selected for the pretraining stage, as these configurations yield the most pronounced increase in overlap ratio.

**Supplementary Fig. 4: Performance of pretrained SinCAA in identifying similar ncAAs compared with randomly sampled ncAAs on the validation set of pretraining ncAAs.** The model achieves AUROC values above 0.996 for identifying the top three most similar ncAAs.

**Supplementary Fig. 5: Statistical overview of the KRAS dataset.** The number of cycles in each peptide was calculated by defining a cycle as a ring structure containing a minimum of eight atoms.

**Supplementary Fig. 6: Performance of various pretraining models on datasets stratified by similarity thresholds to the training data, evaluated using a 2-layer MLP.**

**Supplementary Fig. 7: Spearman’s correlation between SinCAA embedding distance and affinity changes for peptide groups differing at a single position (top), with corresponding example peptide structures highlighting the mutated position (bottom).**

**Supplementary Fig. 8: Comparison of conformational similarity and Morgan similarity in capturing binding affinity changes for peptide groups differing at a single residue position.** Each point represents one peptide substitution group containing variants that differ at a single residue position. For each group, Spearman’s ρ was calculated between residue-pair similarity and (left) pairwise Kd differences among all substitution pairs within the group, or (right) the similarity to the lead peptide and the corresponding Kd difference, using either conformational similarity (y-axis) or Morgan similarity (x-axis). Conformational similarity achieves higher correlations than Morgan similarity for most groups under both evaluation settings, with a more consistent advantage in the lead-centered comparison (right).

**Supplementary Fig. 9: Prediction error for Kd (a) and IC50 (b) with different downstream models stratified by the number of pretrain-unseen ncAAs.** There are 50, 802, and 51 peptides containing 1, 2, and 3 unseen ncAAs, respectively. Across both the Kd and IC50 prediction tasks and all three evaluated downstream model architectures, errors did not increase monotonically with the number of unseen ncAAs. Although this comparison reached significance when using a 2-layer GIN to predict Kd (Kruskal-Wallis H = 10.51, p = 0.005), the group containing only a single unseen ncAA showed the highest error rather than the lowest.

**Supplementary Fig. 10: Micro-F1 scores of various methods for predicting KRAS/NRAS selectivity.**

**Supplementary Fig. 11: UMAP projections of peptide embeddings generated by SimSGT, TGT, and PepLand, with silhouette coefficient (SC) values annotated.**

**Supplementary Fig. 12: Performance of various methods in predicting cell penetration using an MLP (a) and a 1-layer GIN (b).**

**Supplementary Fig. 13: a. Comparison of correlations between the top 20 SinCAA embedding dimensions and experimental cell-penetration scores with those obtained from baseline methods.** Statistical significance was assessed using a one-sided Mann–Whitney U test. SinCAA exhibited stronger correlations than most baseline methods, with Hi-GMAE showing comparable performance. **b. Prediction errors for cell-penetration ability stratified by the number of ncAAs unseen during pretraining.** The test set contains 60, 217, 60 and 7 peptides with 0, 1, 2, and ≥3 unseen ncAAs, respectively. Across the three downstream predictors, two comparisons reached statistical significance. Notably, peptides containing only ncAAs observed during pretraining exhibited the highest or statistically indistinguishable highest mean error, whereas peptides containing unseen ncAAs showed comparable or lower errors.

**Supplementary Fig. 14: Performance of each method in predicting arylomycin-related antibacterial properties.** Assays CHEMBL5523146–CHEMBL5523148, derived from CHEMBL5518248 (68 molecules), correspond to antibacterial activity against Escherichia coli ATCC 35218, E. coli 14-2, and E. coli 14-4, respectively, assessed as inhibition of bacterial growth after 18–20 h using the broth microdilution method. Assays CHEMBL5375618–CHEMBL5375620, derived from CHEMBL5370681 (31 molecules), correspond to inhibition of LepB in E. coli IMP (measured as antibacterial activity via reduction in bacterial growth after 18 h using a two-fold serial dilution method), antibacterial activity against E. coli 25922 (18 h incubation, two-fold serial dilution), and antibacterial activity against E. coli 25922 in the presence of 50% mouse serum (18 h incubation, two-fold serial dilution), respectively. All experimental measurements are reported in μg/mL. For computational analysis, values were transformed by applying a negative logarithm to improve numerical resolution and facilitate model evaluation. The regularization parameter of Ridge regression was fixed at 10 to mitigate overfitting.

**Supplementary Fig. 15: Illustrative optimization of low-permeability peptides using SinCAA-guided substitution screening.** For each of the five peptides with the lowest cell permeability in the test set, residue substitutions were generated by selecting the top-20 conformationally similar ncAA candidates at each position (restricted to the training-set vocabulary). The resulting peptide candidates were scored using the same two-layer GIN permeability prediction model as in Fig. 3, after excluding sequences present in the training set to avoid data leakage. The top three predicted candidates for each starting peptide are shown together with their predicted permeability scores. These candidates have not been experimentally validated and are presented solely to illustrate how SinCAA can be applied to peptide optimization.

**Supplementary Fig. 16: AUROC scores of different methods for predicting peptide binding sites in general protein–peptide interaction tasks.** Results are shown for datasets split using various similarity thresholds and splitting strategies, including novel-protein, novel-peptide, and both-new settings. Due to class imbalance, with binding sites comprising only 33% of all sites, AUROC is presented for reference, while AUPRC offers a more reliable evaluation metric.

**Supplementary Fig. 17: AUPRC score using Hi-GMAE and SinCAA embeddings, evaluated with the same model architecture and training protocol.** SinCAA embeddings consistently outperformed Hi-GMAE embeddings, albeit modestly, likely because most amino acids present in peptides within the PDB dataset are canonical residues.

**Supplementary Fig. 18: Performance comparison for predicting binding sites on proteins.** No substantial improvement was observed when using pretrained peptide embeddings compared with the original PepNN, likely because the protein representations were already sufficiently informative, and the performance on this task had reached saturation.

**Supplementary Fig. 19: Performance comparison of different methods in predicting peptide binding sites for peptides containing ncAAs.** Models were evaluated under three train–test splitting strategies (novel protein, novel peptide, and both new) using sequence similarity thresholds of 0.1, 0.2, 0.3, and 0.4. Across all evaluation settings, SinCAA outperformed all baseline methods.

**Supplementary Fig. 20: Enrichment of high-affinity peptide binders among the top-ranked SinCAA predictions for KRAS structures 7ROV and 8JJS after excluding the highest-scoring predicted binding site outside the experimentally determined peptide-binding pocket.**

**Supplementary Tables**

**Supplementary Table 1: Data extracted from Table 2 of reference 9.**

| Name | Smiles | Kd (nM) |
| --- | --- | --- |
| MeLeu | N([C@@H](CC(C)C)C(=O)O)C | 0.32 |
| MeAla | CN[C@@H](C)C(O)=O | 0.14 |
| MeNva | CN[C@@H](CCC)C(=O)O | 0.75 |
| MeGly(cPent) | CN[C@H](C(=O)O)C1CCCC1 | 0.64 |
| MeThr | CN[C@@H]([C@@H](C)O)C(O)=O | 0.23 |
| MeHse(Me) | CN[C@@H](CCOC)C(O)=O | 0.26 |
| MeSer(Me) | CN[C@@H](COC)C(O)=O | 0.17 |
| MeCha | CN[C@@H](CC1CCCCC1)C(=O)O | 2.6 |
| EtLeu | CCN[C@@H](CC(C)C)C(=O)O | 1.1 |

**Supplementary Table 2: Component ablation results on the KRAS peptide binding dataset.**

| Use ncAAs for pretraining | Number of appended ncAAs in training | Use contrastive learning loss | Layers of GIN in GPS | Test data | Spearman's $\rho$ | | | | | |
| --- | --- | --- | --- | --- | --- | --- | --- | --- | --- | --- |
|  |  |  |  |  | Kd prediction | | | IC50 prediction | | |
|  |  |  |  |  | 2-layer MLP | 1-layer GIN | 2-layer GIN | 2-layer MLP | 1-layer GIN | 2-layer GIN |
| Yes | 1 | Yes | 2 | full test set | 0.573 | 0.755 | 0.759 | 0.599 | 0.725 | 0.718 |
| Yes | 2 | Yes | 2 | full test set | 0.502 | 0.756 | 0.76 | 0.422 | 0.69 | 0.715 |
| Yes | 0 | Yes | 2 | full test set | 0.549 | 0.751 | 0.768 | 0.434 | 0.738 | 0.737 |
| Yes | 0 | Yes | 1 | full test set | 0.409 | 0.715 | 0.738 | 0.309 | 0.688 | 0.71 |
| Yes | 1 | No | 2 | full test set | 0.437 | 0.708 | 0.732 | 0.286 | 0.652 | 0.658 |
| No | - | No | 2 | full test set | 0.368 | 0.717 | 0.726 | 0.421 | 0.674 | 0.666 |
| Yes | 1 | Yes | 2 | test cases with ≥1 unseen ncAA | 0.366 | 0.585 | 0.538 | 0.492 | 0.598 | 0.573 |
| Yes | 2 | Yes | 2 | test cases with ≥1 unseen ncAA | 0.275 | 0.567 | 0.557 | 0.177 | 0.534 | 0.566 |
| Yes | 0 | Yes | 2 | test cases with ≥1 unseen ncAA | 0.401 | 0.552 | 0.578 | 0.234 | 0.577 | 0.577 |
| Yes | 0 | Yes | 1 | test cases with ≥1 unseen ncAA | 0.162 | 0.513 | 0.545 | 0.024 | 0.567 | 0.555 |
| Yes | 1 | No | 2 | test cases with ≥1 unseen ncAA | 0.176 | 0.445 | 0.514 | 0 | 0.472 | 0.502 |
| No | - | No | 2 | test cases with ≥1 unseen ncAA | 0.212 | 0.494 | 0.477 | 0.303 | 0.522 | 0.503 |
